# Profiling Patients with Prolonged Stays in Acute Psychogeriatric Wards in a Tertiary Psychiatric Institution in Singapore

**DOI:** 10.1192/j.eurpsy.2023.548

**Published:** 2023-07-19

**Authors:** T. Hui, S. J. Lee, F. Yao

**Affiliations:** Geriatric Psychiatry, Institute of Mental Health, Singapore

## Abstract

**Introduction:**

The Psychogeriatric Department of the Institute of Mental Health (IMH) in Singapore provides acute inpatient services for elderly patients with severe mental health problems. The average length of stay for inpatients in 2020 was 46 days, which was significantly higher than the ideal length of stay set by the hospital of 21 days. This contributed significantly to healthcare costs and reduced bed capacity for acute admissions from the Emergency Service. Prolonged inpatient stays can lead to physical decompensation including reduced muscle strength, pulmonary capacity and osteoporosis.[i]

[i] Creditor MC. Hazards of hospitalization of the elderly. Ann Intern Med. 1993 Feb 1;118(3):219-23. doi: 10.7326/0003-4819-118-3-199302010-00011. PMID: 8417639.

**Objectives:**

We aimed to profile and identify patients in acute psychogeriatric wards who had prolonged inpatient stays.

**Methods:**

A cross-sectional audit was performed. We used a data collection sheet to capture demographic, social and clinical information of all inpatients residing in the acute psychogeriatric wards on 1^st^ November 2020, whose inpatient stays exceeded one month (over-stayers).

**Results:**

**Table:** 

**Features**		Total	26	%
**Clinical**	**Diagnosis**	Dementia	19	73.1
		Schizophrenia		2
		Depression		4
		Obsessive Compulsive Disorder		1
	**Behavioral difficulties**		18	69.2
	**Activities of Daily Living (ADLs) assistance**		22	84.6
**Social**	**Caregiver burnout**		18	69.2
	**Poor relationships**		3	11.5
	**Inadequate additional caregiver support**		3	11.5

Demographic profile: Out of 57 inpatients (28 male patients and 29 female patients), 26 patients (46%) were over-stayers. Out of these 26 over-stayers, 18 patients (69%) were female and 14 patients (54%) were above age 70.

Clinical profile (n=26): 19 patients (73.1%) were diagnosed with dementia. 18 patients (69.2%) had severe Behavioral and Psychological Symptoms of Dementia (BPSD). 22 patients (84.6%) required assistance in their basic activities of daily living.

Social profile (n=26): Caregivers of 18 patients (69%) were burnt out by patient’s behavior problems, which is commonly seen in caregivers for patients with dementia[ii]. Family members of 3 patients (11.5%) were estranged from them. Caregivers of 3 patients (11.5%) had difficulty engaging additional caregiver support for ADL assistance.

[ii] Reuben DB, Romero T, Evertson LC, Jennings LA. Overwhelmed: a Dementia Caregiver Vital Sign. J Gen Intern Med. 2022 Aug;37(10):2469-2474. doi: 10.1007/s11606-021-07054-3. Epub 2021 Aug 13. PMID: 34389938; PMCID: PMC9360256.

**Conclusions:**

The above profiles enabled the department of Geriatric Psychiatry in IMH to identify elderly patients at risk of prolonged hospital stay at the beginning of their admission and improve the care of these patients to reduce their length of stay.

**Disclosure of Interest:**

None Declared

